# Comprehensive bioinformatics analysis of the characterization and determination underlying mechanisms of over-expression and co-expression of genes residing on 20q in colorectal cancer

**DOI:** 10.18632/oncotarget.20204

**Published:** 2017-08-10

**Authors:** Daojiang Li, Changwei Lin, Miao Chen, Nanpeng Li, Yuheng Du, Chen Su, Chunxing Yang, Ni Gong, Hao Wu, Runliu Wu, Arad Jain, Yi Zhang, Xiaorong Li

**Affiliations:** ^1^ Department of General Surgery, The Third Xiangya Hospital of Central South University, Changsha, Hunan 410013, China; ^2^ Center for Experimental Medicine, The Third Xiangya Hospital of Central South University, Changsha, Hunan 410013, China; ^3^ College of Arts and Science, University of Virginia, Charlottesville, Virginia 22904, The United States of America

**Keywords:** 20q, CNA, co-expression, colorectal cancer, adjacent gene

## Abstract

The Long arm of chromosome 20 (20q) is closely related to the development of colorectal cancer, so identifying the expression profile of genes on 20q through a comprehensive overview is indispensable. In this article, preliminar experimental data, several available databases and bioinformatics tools such as the Cancer Genome Atlas, the Encyclopedia of DNA Elements, the JASPAR database and starBase were combined to analyze the correlation between genes and chromosomal aberrations, microRNA and transcription factors, as well as to explore the expression feature and potential regulative mechanism. The results showed that the most frequently unregulated genes in colorectal cancer arelocated on chromosome 20q, present a significant CNA–mRNA correlation.Furthermore, the genes with mRNA overexpression showed co-expression features and tended to be clustered within the same genomic neighborhoods. Then, several genes were selected to carry out further analysis and demonstrated that shared transcription factors, a conserved bidirectional promoter, and competition for a limited pool of microRNAin the 3’UTR of mRNA may be the underlying mechanisms behind the co-expression of physically adjacent genes.Finally, the databases, Lentivirus shRNA, and qPCR were used to find that these adjacent genes with co-expression cooperatively participated in the same biological pathways associated with the pathogenesis and development of colorectal cancer.

## INTRODUCTION

Chromosomal aberrations are presented in about 85% of colorectal cancer (CRC) patients and occur as non-random events, generally involving losses in 8p, 17p and 18q, and gain in 7p, 7q, 8q, 13q and 20q [1]. Among these, gains of 20q were first found through banding analysis in colorectal cancer [2] and it had been observed >65% of CRCs [3], which suggests that the genes encoded on 20q have a key role in contributing to the phenotype of CRC when overexpressed. Further in-depth research demonstrated that the amplification of 20q was conspicuously complex, not all genes with copy number alterations (CNA or CNV) will alter gene expression, and the synergistic effect of multiple genes was important in tumor development [4, 5], thus it become necessary to explore the relationship between CNA and alteration of gene dosage, as well as the comprehensive expression characteristics of the genes on 20q.

Now,compared to pure experimentation with gene function studies, which focuses on one or several selected genes and biological pathways, bioinformatics and database resources have become an important means to analyze, compare and interpret data from a global scale and macroscopic level [6]. The vast amount of molecular data involving genomes, transcriptomes, proteomes and resources from other biological layers are publicly available [6, 7], So in this study, several available databases, bioinformatics tools, preliminarexperimental data were reasonably combined and employed to analyze the correlation between genes located on 20q, CNA, microRNA, transcription, and so forth. Through this comprehensive comparison method, we were able to explore the expression characteristics and potential regulative mechanisms of these genes, through which we can better understand the molecular basis of how these genes on 20q were involved in CRC, and identify novel genomic targets for therapeutic intervention.

## RESULTS

### The genes located on chromosome 20q were frequently upregulated in colorectal cancer

**Figure 1:**
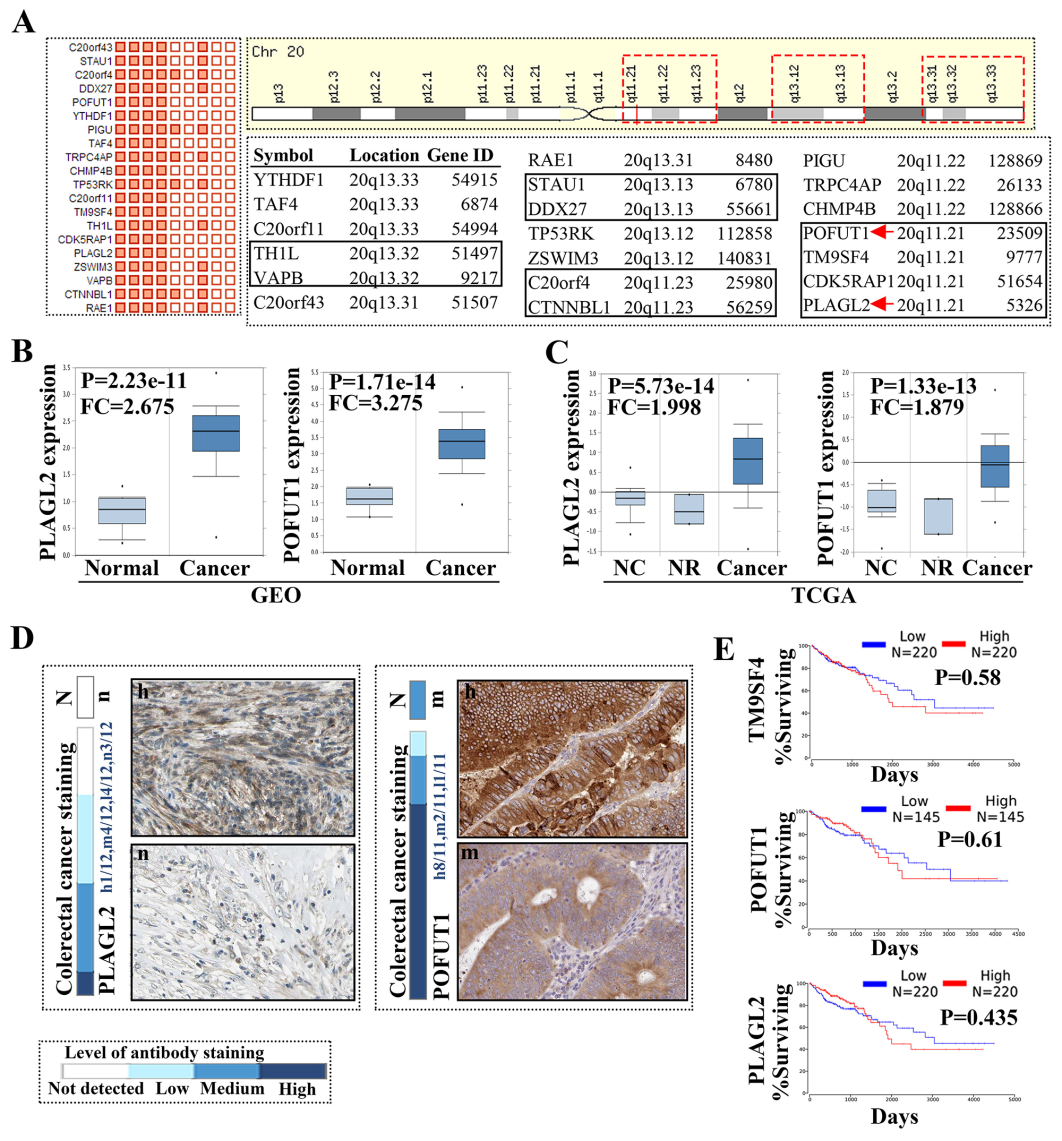
The genes located on chromosome 20q were frequently unregulated in colorectal cancer **(A)** The top 20 genes with mRNA over expression in colon adenocarcinoma (COSMIC study Id: COSU376), the matrix on the left side of the figure list the top unregulated 20 genes in colon cancer (more details can be found in Supplementary Figure 1); the distribution of 20 genes on 20q and the symbol, location and gene ID of each gene were showed on the right side of the figure, red dashed box show all genes located in 3 DNA fragments, black rectangle was used to distinguish the genes located in 20q13.33, 20q13.32, 20q13.31, 20q13.13, 20q13.12, 20q11.23, 20q11.22, 20q11.21. **(B/C)** The representative gene (PLAGL2 and POFUT1) expression in colorectal cancer and normal tissues, which analyzed by Oncomine(GSE9348 and TCGA Colorectal), NC: normal colon, NR: normal rectum, FC: fold change. All other genes demonstrated same result as PLAGL2 (data not show, more detail can be found in Ocomine). **(D)** The representative protein expression derived from The Human Protein Atlas (PLAGL2 and POFUT1 with moderate to strong immunoreactivity was show here, data derived from antibody-based protein profiling using immunohistochemistry), N: normal tissue, n: negative,h: strong or high. **(E)** Survival analysis of PLAGL2, POFUT1 and TM9SF4 in colorectal cancer, data derived from TCGA.

Once the somatic mutations of all genes in colon adenocarcinoma were analyzed by COSMIC, the result showed that the majority of top 20 genes with somatic mutations are mRNA over expression (18/20) and CNA gain, except 2 genes (PCLO with high methylation and APC with high point mutation). When the somatic mutations was only restricted to “Gene expression”, we found that the top 20 genes mainly presented mRNA overexpression and CNA gain, and all were located on 20q, as shown through NCBI and Ensemble identification (Supplementary Figure 1). Further analysis through Map Viewer showed that these genes distributed on 3 adjacent DNA fragments, including 20q13.33, 20q13.32, 20q13.31, 20q13.13, 20q13.12, 20q11.23, 20q11.22 and 20q11.21 (Figure 1A and Supplementary Figure 1). Oncomine was used to confirm gene expression, as compared to normal tissue.TCGA and GEO data all demonstrated that these genes have higher expression in colorectal samples (Figure 1B/1C). Then we used The Human Protein Atlas to clarify the protein expression in colorectal cancer. tThe results revealed that protein showed different cytoplasmic or nuclear immunoreactivity. Proteins such as TM9SF4 and POFUT1 have strong positivity but CTNBL1 has a weak to moderate immunoreactivity and PIGU presented negatively (Figure 1D), which is in line with the fact that mRNA level increases do not translate into increased abundance of the corresponding proteins [8]. Finally, we selected 3 genes with high expression mRNA and protein to carry out survival analysis and found these genes didn’t decrease the survival rate of colorectal patients (Figure 1E).

### The genes located on chromosome 20q that are frequently unregulated in colorectal cancer showed significant CNA–mRNA correlation

**Figure 2:**
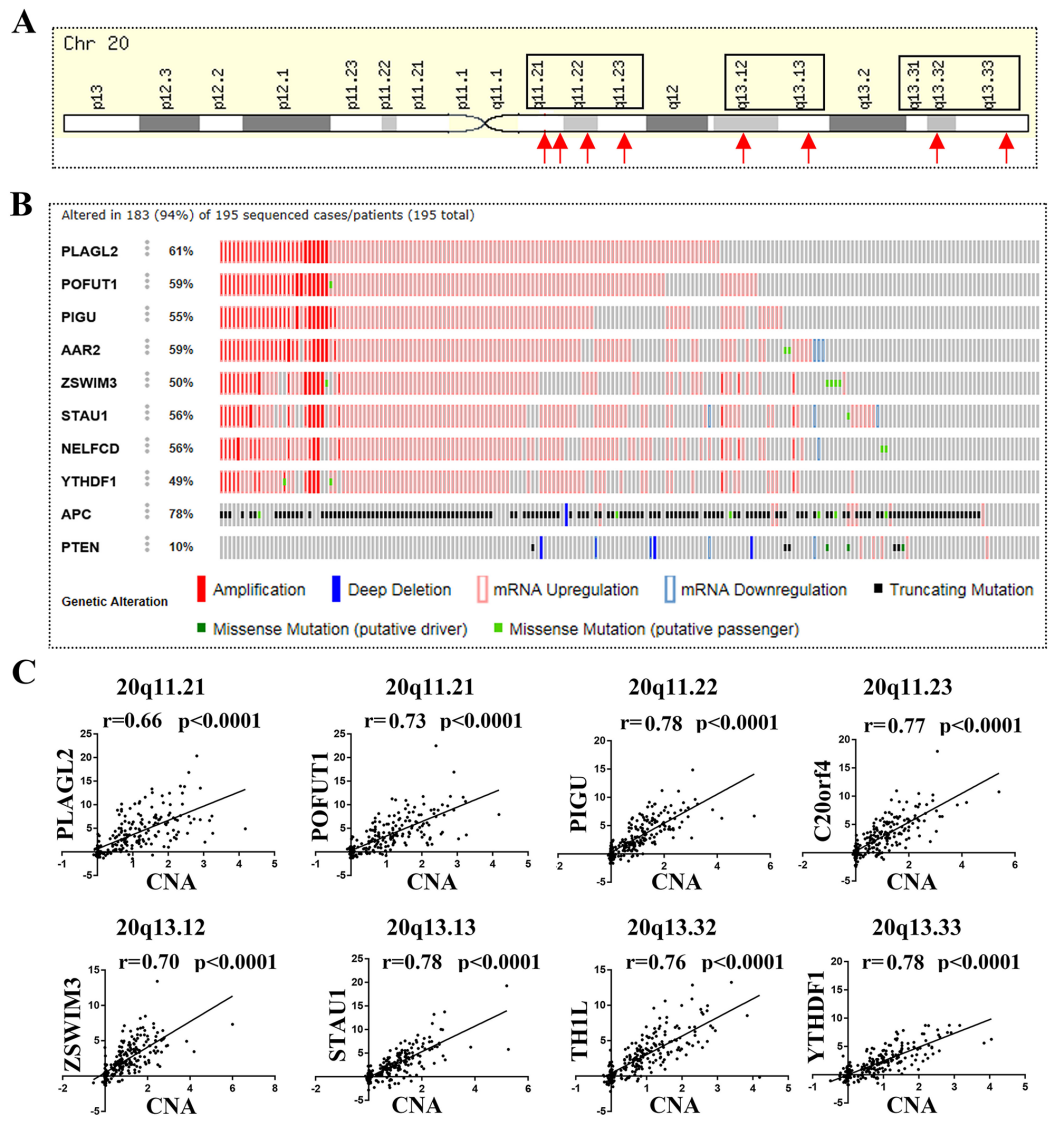
8 genes located on chromosome 20q and frequently unregulated in colorectal cancer showed significant CNA–mRNA correlation **(A)** 8 unregulated genes located on 20q13.33 (YYHDF1), 20q13.32 (NELFCD or TH1L), 20q13.13 (STAU1), 20q13.12 (ZWIM3), 20q11.23 (AAR2 or C20orf4), 20q11.22 (PIGU) and 20q11.21 (PLAGL2 and POFUT1) were selected for somatic mutations analysis in CRC. **(B)** Amplification and mRNA up regulation are the main mutations of 8 genes; the result is evaluated by “The cBioPortal for Cancer Genomics database (bowel-colorectal adenocarcinoma)”. **(C)** 8 genes showed significant CNA–mRNA correlation (p<0.0001, r>0.65).

**Figure 3:**
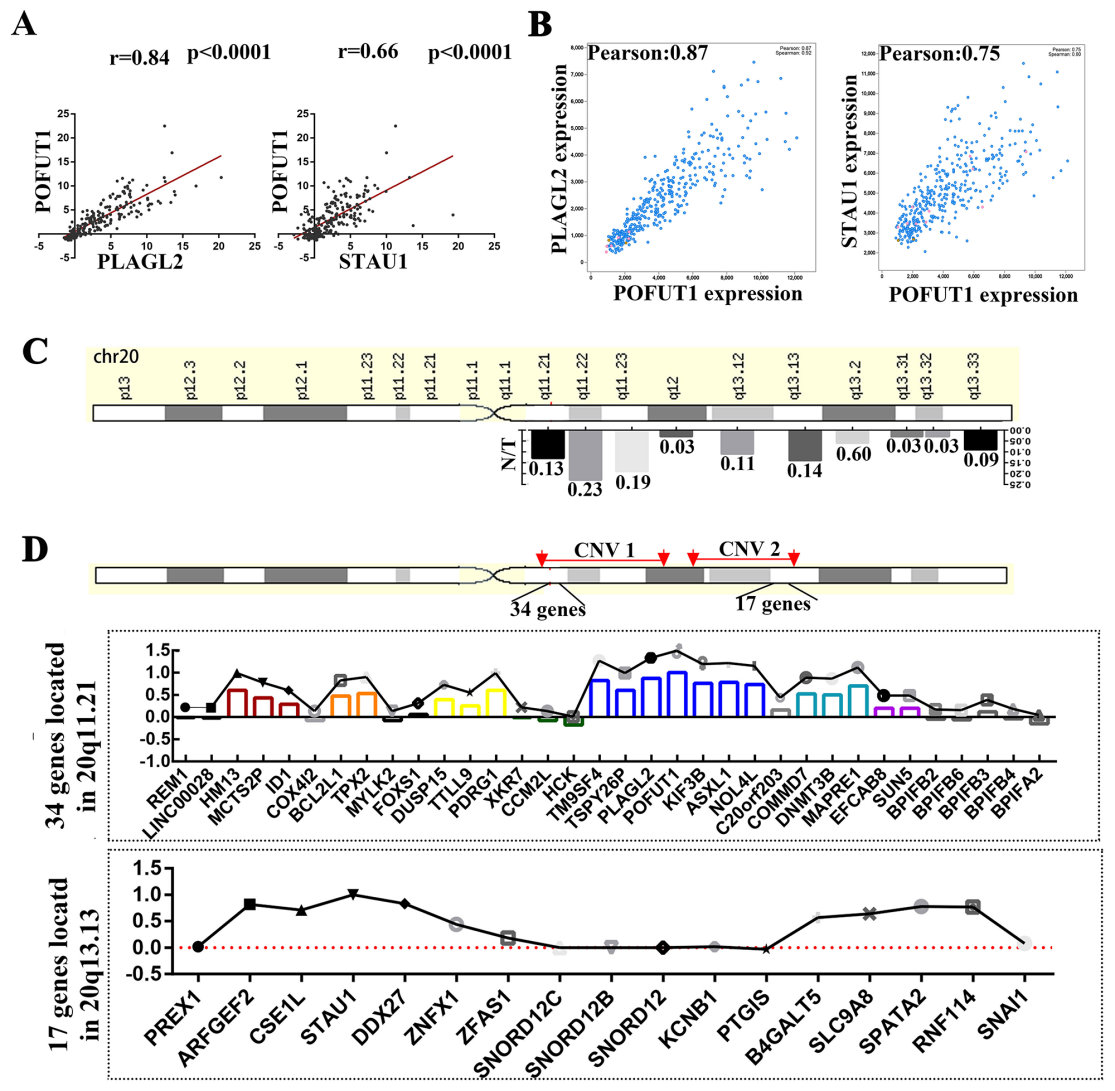
The genes located on chromosome 20q with similar expression levels tend to be clustered within the same genomic neighborhoods **(A)** The mRNA expression of POFUT1 and PLAGL2 or STAU1 shows extremely obvious positive correlation, 218 TCGA sample data with quantifiable mRNA measurements (Supplementary Table 1) were used for mRNA–mRNA evaluation. **(B)** The co-expression data between all genes (20436 genes) and STAU1 and POFUT1 in CRC was download from the cBioPortal for Cancer Genomics (Colorectal Adenocarcinoma, 633 samples) and show same obvious positive correlation between POFUT1 and PLAGL2 or STAU1. **(C)** 86 genes correlated significantly with POFUT1 (Pearson score≥0.6), 96.5% (83/86) gene distributed on 20q and more genes located on 20q11.22,20q11.21 and 20q11.23 (23%, 13% and 19%, respectively, calculated through gene with Pearson score≥0.6/all genes located on this DNA region). **(D)** 34 genes adjacent with POFUT1 and 17 genes adjacent with STAU2 presented a distinct discontinuous distribution and are clustered into co-regulated groups of 1-7 genes such as POFUT1 co-expressed with its adjacent genes TM9SF4(0.82), PLAGL2(0.87), KIF3B(0.76) and ASXL1(0.78), STAU1 co-expressed with ARFGEF2(0.82),CSE1L(0.71) and DDX27(0.83) (gene cluster presented with different color); the ordinate means Pearson score(genes vs. POFUT1 and POFUT1 was set to 1 in the above diagram, genes vs. STAU1 and STAU1 was set to 1 in the image below), CNV1 and CNV2 means two groups of genes located on same focal amplification fragment. (More detail can be found in Supplementary Table 2)

As mentioned above, we found that the top 20 genes with frequently unregulated in colorectal cancer all resided on the long arm of chromosome 20 (20q), In particular these genes with mRNA over expression were accompanied by CNA gain, which remind us DNA amplification may be the reason primary for gene mRNA overexpression. To illustrate this, as showed in Figure 2A, we first selected 8 unregulated genes located on 20q13.33 (YYHDF1), 20q13.32 (NELFCD or TH1L), 20q13.13 (STAU1), 20q13.12 (ZWIM3), 20q11.23 (AAR2 or C20orf4), 20q11.22 (PIGU) and 20q11.21 (PLAGL2 and POFUT1) for an additional somatic mutations analysis in CRC through cBioPortal (Colorectal Adenocarcinoma (TCGA, Provisional) 633 samples). The results showed that Amplification and mRNA overexpression are the main mutations of 8 genes (Figure 2B). Then we downloaded the TCGA data with quantifiable CNA and mRNA measurements for CNA-mRNA correlation analysis, in which the 8 genes showed significant CNA–mRNA correlation (p<0.0001, r>0.65) (Figure 3C).

### The genes with mRNA overexpression on 20q exhibited significant co-expression and tended to be clustered within the same genomic neighborhoods

**Figure 4:**
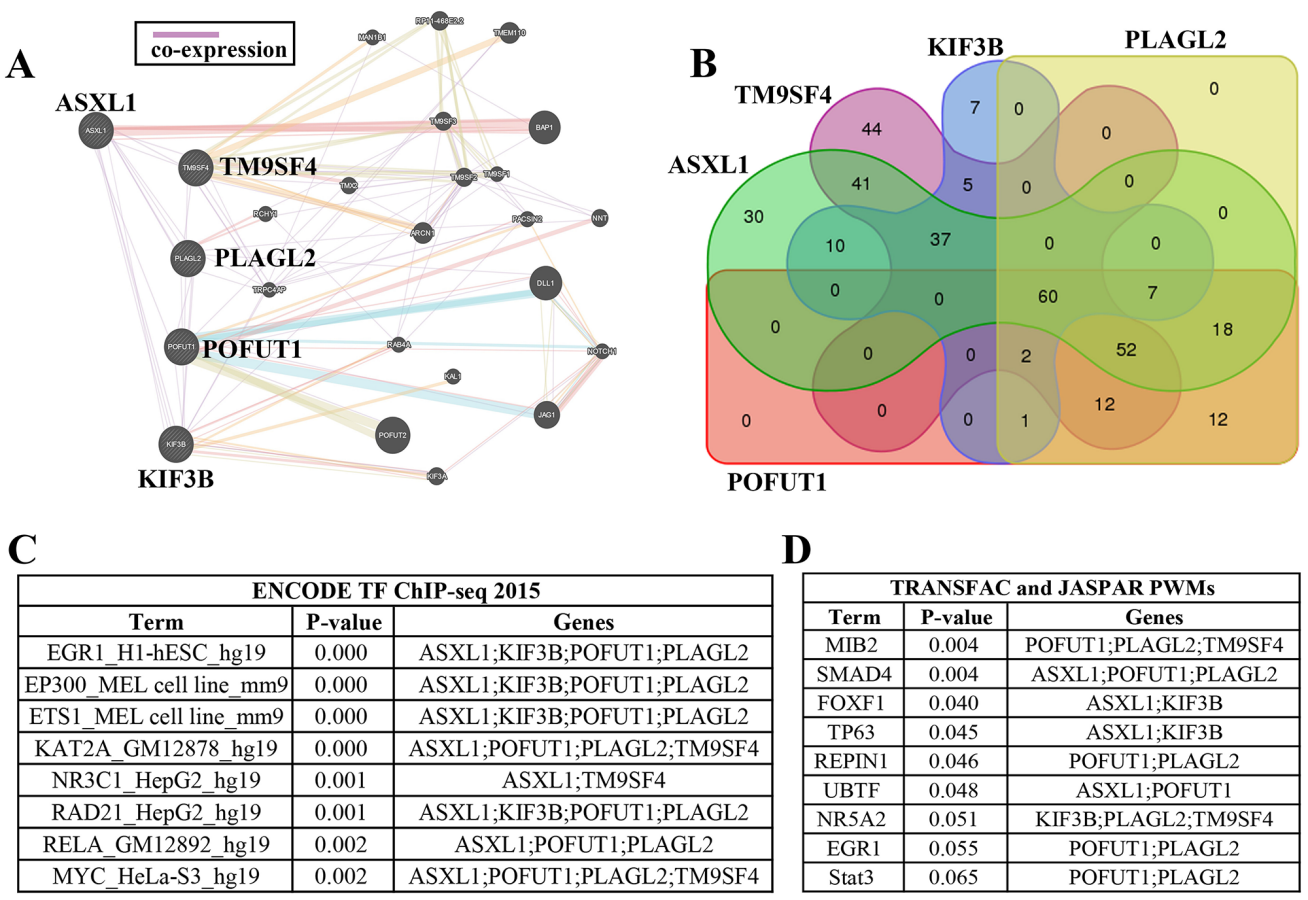
Adjacent genes with co-expression may be regulated in part by common transcription factors **(A)** Interaction networks predicted by GeneMANIA also identified TM9SF4, POFUT1, PLAGL2, KIF3B and ASXL1 showed co-expression. **(B)** Venn diagrams indicated that the promoters of these 5 genes share 60 transcription factors supported by ChIP-Seq evidence (the transcription factors of each gene and intersections can be found in Supplementary Table 3). **(C)** The representative enrichment analysis results for ENCODE gene-set library found that 2-4 adjacent genes may share same transcription factor (more details can be found in Supplementary Table 3). **(D)** The representative enrichment analysis results for PWMs from TRANSFAC and JASPAR gene-set library found that 2-4 adjacent genes may share same transcription factor (more details can be found in Supplementary Table 3). C and D analyzed by software Enrichr.

Interestingly, further analysis of 3 genes (PLAGL2, POFUT1and STAU1) located on chromosome 20q and frequently unregulated in colorectal cancer exhibited significant co-expression levels (PLAGL2 vs. POFUT1, r=0.84, p<0.0001; POFUT1 vs.STAU1, r=0.66, p<0.0001), which prompted us to explore the co-expression of all genes located on 20q (Figure 3A). 2 genes,STAU1 and POFUT1,which are ranked 2 and 5 respectively in all genes with mRNA overexpression in CRC (Supplementary Figure 1 and Figure 1A),were selected for this study. For the convenience of calculations and comparable statistic results (Figure 3B), the co-expression data between all genes (20436) and STAU1 and POFUT1 in CRC was downloaded from The cBioPortal for Cancer Genomics (Colorectal Adenocarcinoma (TCGA, Provisional) 633 samples) (Supplementary Table 2). Firstly, the data revealed that there are 86 genes correlated significantly with POFUT1 (pearson Score≥0.6), 96.5% (83/86).These genes are uniformly distributed on 20q with more genes located on 20q11.22, 20q11.21 and 20q11.23 (23%, 13% and 19%, respectively).( calculated through “gene with pearson Score≥0.6”/“all genes located on this DNA region”) (Figure 3C and Supplementary Table 2). It is noteworthy that 20q11.22, 20q11.21 and 20q11.23 and POFUT1 (20q11.21) are within genomic neighborhoods. We then wanted to explore whether the genes correlated significantly with POFUT1 and STAU1 also tend to lie adjacent to each other in the genome. 50 genes with same CNA and located adjacent to each other in the 20q11.21 (POFUT1) and 20q13.13 (STAU1) were selected for study, as demonstrated in Figure 3C/3D and Supplementary Table 2. 34 genes on 20q11.21 and 17 genes on 20q13.13 remained for analysis and the results found that the genes correlated significantly with POFUT1 and STAU1 have a distinct disjunctive distribution and are clustered into co-regulated groups of 2-7 genes.For example, POFUT1 co-expressed with its adjacent genes TM9SF4(0.82), PLAGL2(0.87), KIF3B(0.76) and ASXL1(0.78), STAU1 co-expressed with ARFGEF2(0.82),CSE1L(0.71) and DDX27(0.83). In addition, interaction networks predicted by GeneMANIA also support this conclusion (Figure 4A).

### Shared transcription factors in promoter sequences may be one mechanism contributing to co-expression of adjacent genes

Previous studies have discovered that a large proportion of transcriptome in human is linearly arranged in small groups of adjacent genes which tend to be regulated by the same transcription factors [9], so the adjacent gene group including TM9SF4, POFUT1, PLAGL2, KIF3B and ASXL1 was arranged for further study. Venn Diagrams indicated that the promoters of these 5 genes share 60 transcription factors which was supported by ChIP-Seq evidence (Figure 4B, Supplementary Table 3). In addition, the co-expression analysis between these shared transcription factors and POFUT1 was carried out using co-expression data derived from cBioPortal, The results showed 55% (33/60) presented Pearson Score≥0 such as TGIF2(0.64), myc(0.43) and SP3(0.32); 45%(27/60) presented Pearson Score≤0 such as RCOR1(-0.38), SOX13(-0.37) and SP1(-0.19) (Supplementary Table 3),indicating that which indicated POFUT1 can be activated or repressed by the transcription factors as discussed above. Then we performed gene set enrichment analysis by means of Enrichr software. Two gene-set libraries about the transcription category were used in this article ( PWMs from TRANSFAC and JASPAR, The ENCODE transcription factor gene-set). The enrichment analysis results for the ENCODE gene-set library found that 2-4 adjacent genes may share same the transcription factor, such as ASXL1,KIF3B, POFUT1 and PLAGL2,which shareEGR1(p<0.0001)(Figure 4C and Supplementary Table 3), However analysis of PWMs from TRANSFAC and JASPAR gene-set libraria revealed that 2-3 genes may share the same transcription factors, such as ASXL1,POFUT1 and PLAGL2 which share SMAD4(p=0.004) (Figure 4D and Supplementary Table 3).

### Gene pair regulated by evolutionarily conserved bidirectional promoter may be a classic mechanism explaining co-expressions of physically adjacent genes

**Figure 5:**
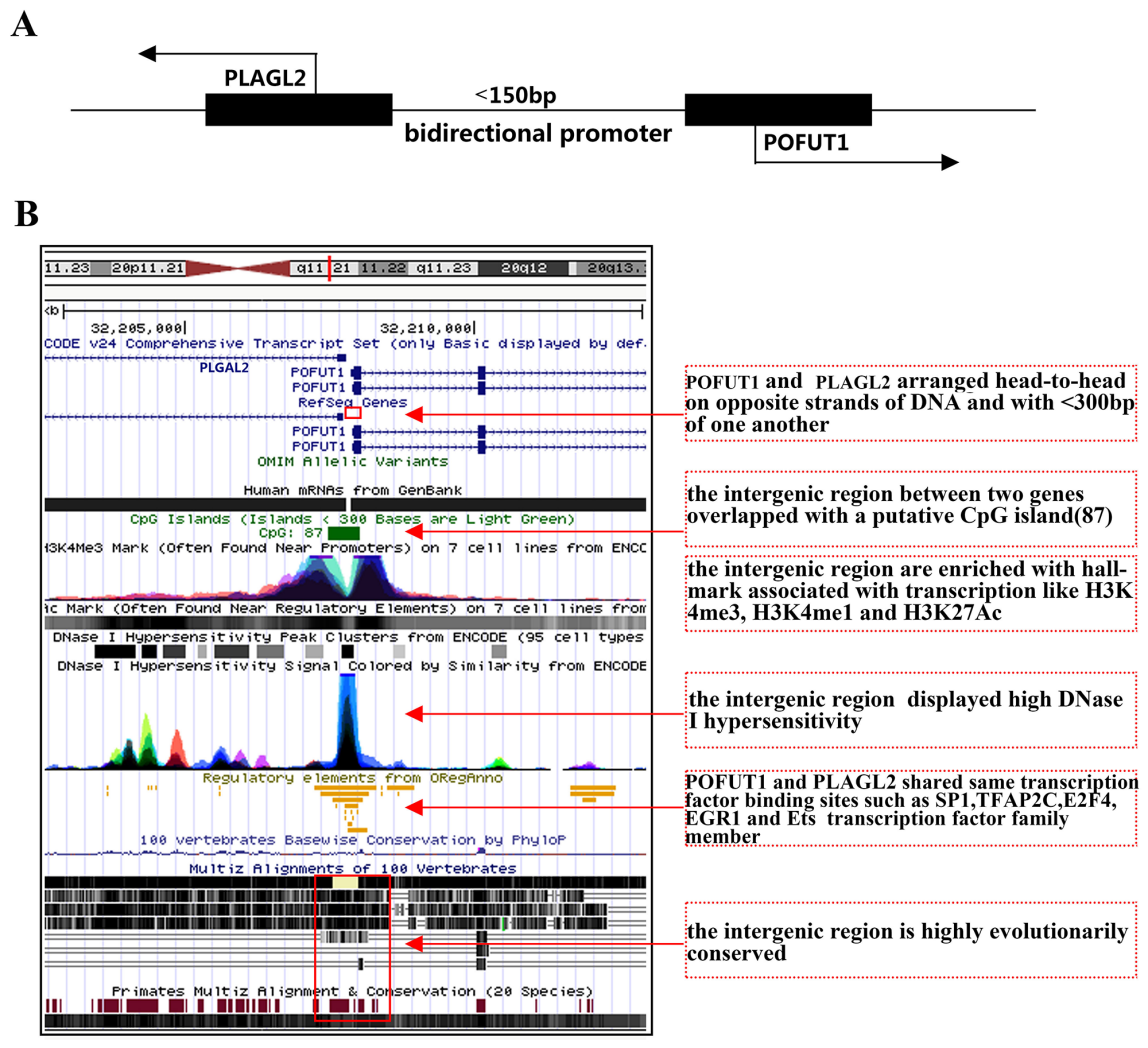
PLAGL2 and POFUT1 are classic bidirectional gene pair **(A)** Schematic representation of a bidirectional promoter between PLAGL2 and POFUT1 genes, black arrows denote transcription direction of both genes. **(B)** We employed UCSC genome browser(alter tracks displayed) to analyze the genomic organization, the human PLAGL2 and POFUT1 are located on 20q11.21 in a head-to-head orientation, the start of both genes are separated less than 150bp and overlapped with a putative CpG island (87bp), according to ENCODE v24 annotation determined by a ChIP-seq assay, the intergenic region displayed high DNase 1 hypersensitivity and was enriched with histone hallmark associated with transcription like H3K4me3, H3K4me1 and H3K27Ac, furthermore the nucleotide sequence highly evolutionarily conserved (more details can be found in Supplementary Table 4) and contains multiple potential DNA motifs including SP1,TFAP2C, E2F4, EGR1 and Ets transcription family, which have been verified overrepresented in bidirectional promoter.

**Figure 6:**
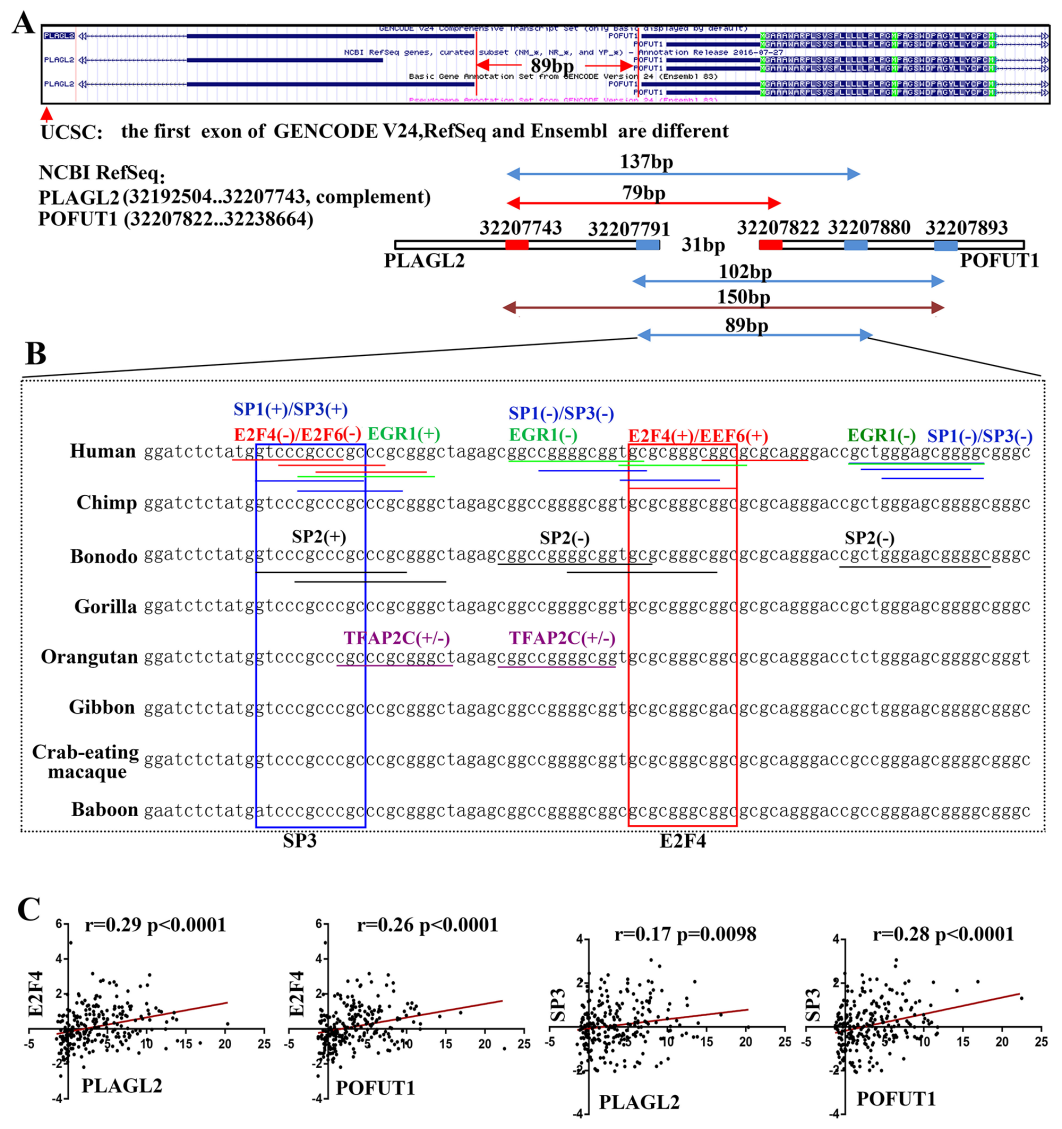
The optimal bidirectional promoter and transcription factor **(A)** Because 3 gene database including NIBI RefSeq, ENCODE v24 and Ensembl genome browser 87 displayed different positionfor PLAGL2 and POFUT1, so we draw a schematic diagramto find out the optimal nucleotide sequence (89bp between32207791 to 32207880) for bidirectional promoter, two-way arrow denote the base number between the different start of both genes (the start derived from NIBI RefSeq was marked red and show here, and more descriptions can be found in Supplementary Table 4). **(B)** Base pair alignment between human and several other species in the region between 32207791 to 32207880 (89bp), the predicted transcriptions factor binding sites are marked by horizontal line with different color (SP1 and SP3 with blue, SP2 with black, E2F4 and E2F6 with red, EGR1with green, TFAP2C with purple). Two features have been observed, first and foremost, the base sequence including transcription factor binding sites are highly conserved during evolution, besides all predicted transcription factor binding sites mainly focused on 3 regions, especially TFAP2C can better binding to positive-sense(+) and negative-sense(-) strand. Among these transcriptions factor, E2F4 and SP3 were selected for co-expression analysis and labeled with rectangular box. (More conserved nucleotide sequence and details can be found in Supplementary Table 4 and transcriptions factor binding sites analyzed by The JASPAR database can be found in Supplementary Figure 2.) **(C)** Transcriptions factor E2F4 and SP3 correlated significantly with POFUT1 and PLAGL2 in colon and rectal cancer.

As analyzed above, what is particularly intriguing in enrichment analysis of transcription factor is that gene pair, PLAGL2 and POFUT1, was the shortest physical sequence distance (<150bp) and are the most common 2 genes shared transcription factor. More importantly, the mRNA expression of PLAGL2 correlated significantly with that of POFUT1(r=0.87,p<0.0001, Figure 3A) and Venn Diagrams in Figure 4B showed PLAGL2 and POFUT1 shared all 164 transcription factors based on ChIP-Seq evidence (Supplementary Table 3). Thus, the PLAGL2 and POFUT1 pair was selected as a candidate for further study. Firstly, we employed UCSC genome browser to analyze the genomic organization. Interesting, the results suggest the human PLAGL2 and POFUT1are located on 20q11.21 in a head-to-head orientation, with the start of both genes are separated less than 150bp and overlapped with a putative CpG island. According to ENCODE v24 annotation, the intergenic region displayed high DNase 1 hypersensitivity and was enriched with hallmarks associated with transcription like H3K4me3, H3K4me1 and H3K27Ac. Furthermore, the region lacks TATA box, is highly evolutionarily conserved (Supplementary Table 4), and contains multiple potential DNA motifs including SP1,TFAP2C, E2F4, EGR1 and Ets transcription families, which have been verified to be overrepresented in the bidirectional promoter [10] (Figure 5A/5B). These result strongly indicatethat PLAGL2 and POFUT1 are a classic bidirectional gene pair. Next, as shown in Figure 6A, 89 bp nucleotide sequences, which regard as optimal bidirectional promoter by computer-assisted analyses, was retained for more thorough analysis. The results of this demonstrated that this sequence was highly evolutionarily conserved and contains multiple transcription factor binding sites, including SP1, SP3, SP2, E2F4, E2F6, EGR1and TFAP2C (Figure 6B and Supplementary Figure 2). Finally, 244 TCGA sample data sets with quantifiable mRNA measurements (Supplementary Table 4) were used for mRNA (transcription factor)-mRNA correlation evaluation. The results showed that E2F4 and SP3 correlated significantly with POFUT1 and PLAGL2 in colon and rectal cancer (Figure 6C), which indicated E2F4 and SP3 are the most likely transcription factor binding to this bidirectional promoter and causing co-expression of the gene pair.

### Competition for a limited pool of microRNAs mediates cross-regulation of mRANs may be another mechanism contributing to the co-expression of adjacent genes

**Figure 7:**
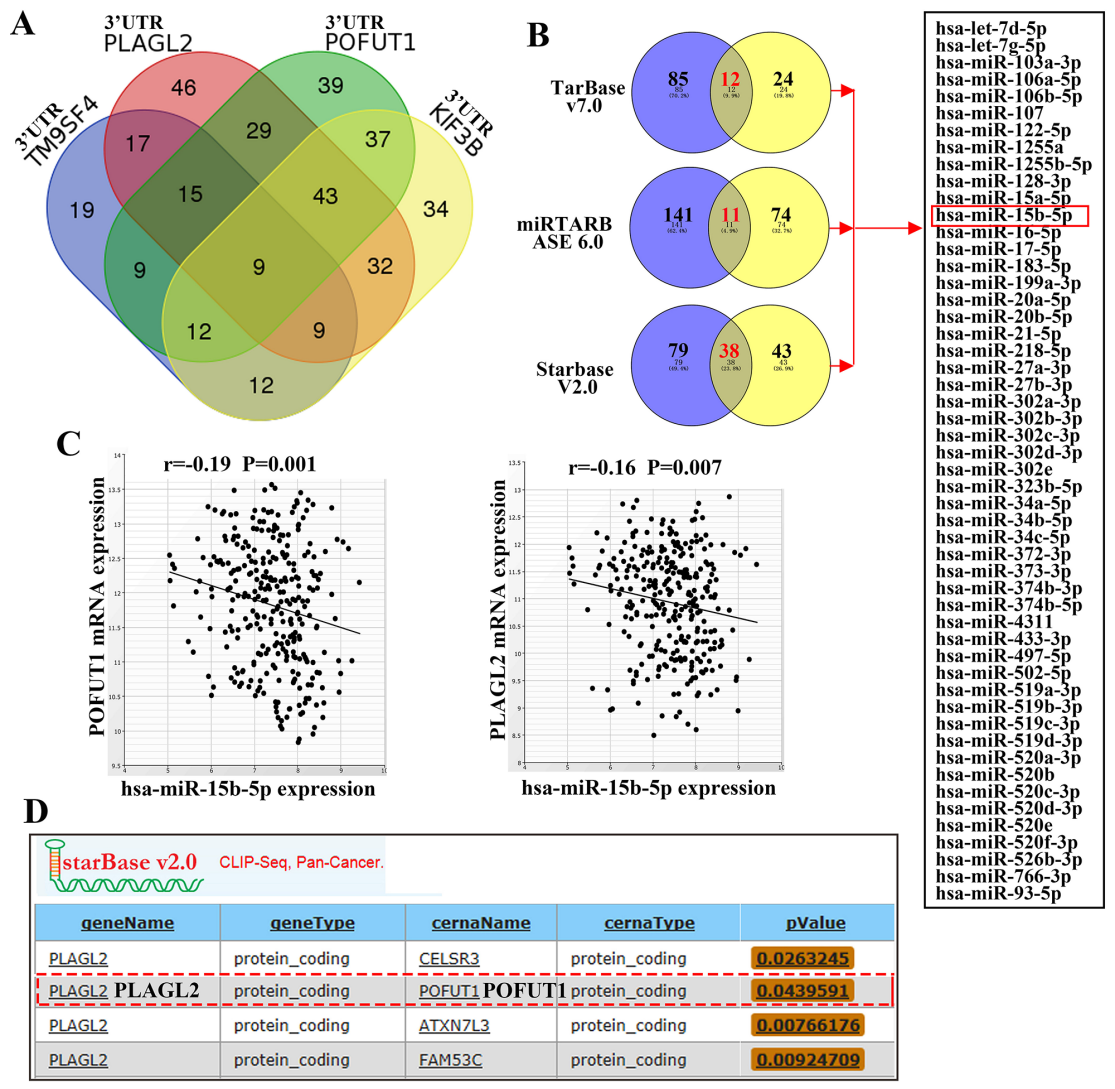
Adjacent genes with co-expression, especially adjacent gene pair, may be mediated by ceRNA **(A)** Venn Diagrams demonstrated that 3’UTR of five genes shared 9 miRNAs predicted by TargetScan. **(B)** The intersections of experimentally validated microRNAs of PLAGL2 and POFUT1 are 12 and 11 in TarBase v7.0 and miRTARBASE6.0, and the intersections of microRNAs in Starbase v2.0 is 38, the union of 3 intersections is 52, which indicated both genes share common 52 microRNAs with experimental evidence. **(C)** The representative anti-correlation (pearson correlation: r<0, p-value<0.05) between miRNA and target genes across colon and rectal cancer. **(D)** Starbase v2.0 predicted that POFUT1 is a perfect ceRNA of PLAGL2. (More details about Figure 7 can be found in Supplementary Table 5.)

Apart from the regulation of transcription in nucleus, a number of recent studies unraveled that co-expression of mRNA may be caused by the competing endogenous RNA (ceRNA) mechanism in cytoplasm. The 3’UTR of different mRNA transcripts containing common microRNA recognition elements(MRES) can compete for common microRNAs, by which different mRNA transcripts can keep co-expressing [11]. So in this article, we explore the possibility that co-expression of adjacent genes can be brought out by a ceRNA mechanism. To start with, the adjacent gene group including TM9SF4, POFUT1, PLAGL2, KIF3B and ASXL1 was arranged for this study. Venn Diagrams demonstrated that these five genes shared 9 miRNAs predicted by TargetScan (Figure 7A).Then, considering adjacent gene pairs may share most micRNAs than beyond 3 genes, POFUT1 and PLAGL2 wereselected for further study. The intersections of experimentally validated microRNAs of both genes are 12 and 11 in TarBase v7.0 number of and miRTARBASE6.0. In addition, we found the number of intersections of microRNAs in Starbase v2.0, which was processed from five miRNA prediction software programs (TargetScan, PicTar, PITA, miRanda and RNA22) and overlapped with CLIP-Seq data, is 38. As showed in Figure 7B, Figure 7C and Supplementary Table 5, PLAGL2 and POFUT1 share common 52 microRNAs as shown through experimental evidence. Furthermore these microRNAs can directlay target PLAGL2 and POFUT1 and repress the expression of both genes in colon and rectal cancer. All these results and ceRNA prediction by Starbase v2.0 (Figure 7D) suggested that the adjacent genes with co-expressionmay be mediated by ceRNA.

### Co-expression of adjacent genes cooperatively participate in the same biological pathways associated with the pathogenesis and development of colorectal cancer

**Figure 8:**
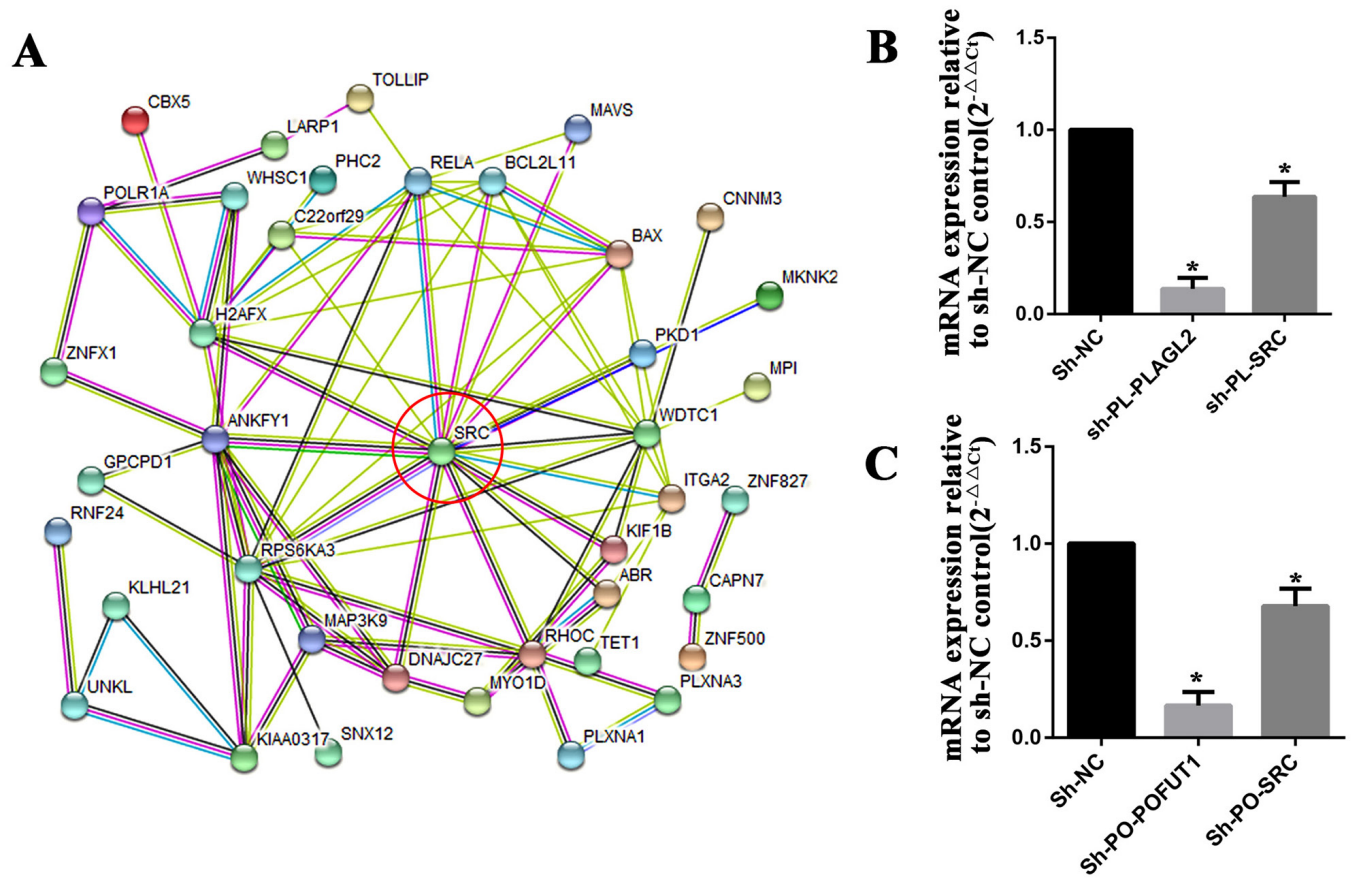
PLAGL2 and POFUT1 cooperatively participated in SRC biological pathways **(A)** 63 genes which can maintain correlation with POFUT1and PLAGL2 in 14 human tumor tissues were selected for protein-protein interactions by STRING v10.5, the oncogenes SRC is the most important intersection point. **(B)** The knock-out of gene PLAGL2 (sh-PL-PLAGL2) in colorectal cancer cell SW620 led to inhibition of SRC (sh-PL-SRC). **(C)** The knock-out of gene POFUT1 (sh-PO-POFUT1) in colorectal cancer cell SW620 led to inhibition of SRC (sh-PL-SRC).

As discussed above, a paires of adjacent genes with co-expression were selected for further analysis which showed a sharing of transcription factors, an evolutionary conserved bidirectional promoter and/or competition for a limited pool of microRNAs may be the underlying mechanism leading to co-expression, but this can only explain a part of the phenomenon. More research needs to be done to clarify the biological process. Compared with the difficulty in interpreting the co-expression mechanism, what was really noticeable was that the biological pathways that these genes are involved in are very similar. 6 adjacent genes with co-expression characterization as shown in Figure 3(TM9SF4, PLAGL2, POFUT1, KIF3B, ASXL1, NOL4L) were chosen for gene-set pathway enrichment analysis. We first carried out KEGG pathway enrichment analysis for each of the 6 genes.The gene-set of each gene (Pearson CorrelationCoefficient≥0.2, Supplementary Table 8) was compiled from cBioPortal for Cancer Genomics (Colorectal Adenocarcinoma (TCGA, Nature 2012) 276 samples), then a Venn analysis was performed to calculate the statistically significant intersection(s) of KEGG pathways of each gene. As shown in Table 1, 6 adjacent genes share common biological pathway such as “Endometrial cancer”,“Hippo signaling pathway”,“Choline metabolism in cancer”,“Pathways in cancer”,“Signaling pathways regulating pluripotency of stem cells”,“Wnt signaling pathway”, and“Adherens junction”. Among these, TM9SF4, POFUT1 and KIF3B are all involved in the pathway of “Colorectal cancer”. All indicated that co-expression of adjacent genes cooperatively participated in the same biological pathways associated with the pathogenesis and development of human cancers, including colorectal cancer; furthermore these adjacent genes maintain co-expression through various mechanisms, are may be designed to participate in the common function. In addition, PLAGL2 and POFUT1, which are separated by shortest physical sequence distance (<150bp) and exhibit co-expression characteristics in 14 cancer types (Supplementary Table 7) were used for further analysis. 63 genes(Stable7) which can maintain correlation with POFUT1and PLAGL2 in a variety of human tumor tissues were selected for protein-protein interactions by STRING v10.5, as shown in Figure 8A. The oncogenes, *SRC* is the most important intersection point, so we inhibited the expression of PLAGL2 and POFUT1 in SW620 cells.The results found that low-expression of *SRC* can be found in two knockout cells (Figure 8B), which indicate PLAGL2 and POFUT1 cooperatively participate in *SRC* biological pathways, and therefore promote pathogenesis and development of colorectal cancer.

**Table 1:** The Venn result of KEGG pathway of 6 genes with co-expression and tending to be clustered within the same genomic neighborhoods

Names	Total	Elements
ASXL1 KIF3B NOL4L PLAGL2 POFUT1 TM9SF4	10	hsa05213:Endometrial cancer
		hsa04390:Hippo signaling pathway
		hsa04910:Insulin signaling pathway
		hsa05231:Choline metabolism in cancer
		hsa05200:Pathways in cancer
		hsa04919:Thyroid hormone signaling pathway
		hsa04152:AMPK signaling pathway
		hsa04550:Signaling pathways regulating pluripotency of stem cells
		hsa04310:Wnt signaling pathway
		hsa04520:Adherens junction
ASXL1 KIF3B PLAGL2 POFUT1 TM9SF4	1	hsa05217:Basal cell carcinoma
ASXL1 KIF3B NOL4L POFUT1 TM9SF4	5	hsa04931:Insulin resistance
		hsa00310:Lysine degradation
		hsa04012:ErbB signaling pathway
		hsa04144:Endocytosis
		hsa05215:Prostate cancer
ASXL1 KIF3B NOL4L PLAGL2 POFUT1	1	hsa05169:Epstein-Barr virus infection
KIF3B PLAGL2 POFUT1 TM9SF4	2	hsa03320:PPAR signaling pathway
		hsa04122:Sulfur relay system
ASXL1 NOL4L PLAGL2 TM9SF4	1	hsa04360:Axon guidance
KIF3B NOL4L POFUT1 TM9SF4	1	hsa05219:Bladder cancer
ASXL1 KIF3B NOL4L POFUT1	1	hsa03015:mRNA surveillance pathway
NOL4L PLAGL2 TM9SF4	2	hsa04015:Rap1 signaling pathway
		hsa04340:Hedgehog signaling pathway
KIF3B POFUT1 TM9SF4	1	hsa05210:**Colorectal cancer**
ASXL1 NOL4L TM9SF4	3	hsa04530:Tight junction
		hsa04120:Ubiquitin mediated proteolysis
		hsa03018:RNA degradation
KIF3B PLAGL2 POFUT1	3	hsa04975:Fat digestion and absorption
		hsa03020:RNA polymerase
		hsa04146:Peroxisome
ASXL1 KIF3B POFUT1	3	hsa03008:Ribosome biogenesis in eukaryotes
		hsa04110:Cell cycle
		hsa03013:RNA transport
KIF3B NOL4L POFUT1	1	hsa04068:FoxO signaling pathway
PLAGL2 TM9SF4	3	hsa00561:Glycerolipid metabolism
		hsa04916:Melanogenesis
		hsa04976:Bile secretion
ASXL1 TM9SF4	2	hsa04350:TGF-beta signaling pathway
		hsa04710:Circadian rhythm
NOL4L TM9SF4	9	hsa05223:Non-small cell lung cancer
		hsa04911:Insulin secretion
		hsa04330:Notch signaling pathway
		hsa04666:Fc gamma R-mediated phagocytosis
		hsa05211:Renal cell carcinoma
		hsa04070:Phosphatidylinositol signaling system
		hsa05220:Chronic myeloid leukemia
		hsa05205:Proteoglycans in cancer
		hsa05120:Epithelial cell signaling in Helicobacter pylori infection
KIF3B POFUT1	6	hsa00240:Pyrimidine metabolism
		hsa00072:Synthesis and degradation of ketone bodies
		hsa01100:Metabolic pathways
		hsa00230:Purine metabolism
		hsa00600:Sphingolipid metabolism
		hsa04964:Proximal tubule bicarbonate reclamation

The gene-set of each gene (Pearson correlation coefficient≥0.2, Supplementary Table 8) was compiled from cBioPortal for Cancer Genomics (Colorectal Adenocarcinoma (TCGA [36]) 276 samples).

## DISCUSSION

The chromosome 20q, as first observed by means of banding analysis in colorectal cancer, frequently exhibits complex copy number amplification patters that have been reported in a variety of other cancer [2, 12]. Further study thereafter showed gains in 20q occurs in >65% of CRCs, the most significant of which were mostly found in 20q11 and 20q13, Patients in 20q gain were always been associated with poor prognoses [1, 13]. Previous studies have confirmed that CNA plays an important role in the altering expression of genes residing on it. A few example include amplification of the MYC oncogeneof 8q24 resulting in its over-expression, assuming a role a central driver in CRCs [14], the amplification of 20q, which has been reported causing mRNA overexpression of AURKA (20q13.2) and TPX2 (20q11), ultimately promoting progression from colorectal adenoma to carcinoma [13]. Integrated analysis of copy number variation and genome-wide expression profiling in CRC identified 56 overlapping genes,and 48 of which (85.7%) showed a positive association [15]. Similar conclusions were obtained in our study including two aspects. For one thing, the top 20 genes with mRNA overexpression in CRC are all located on 20q and significantly correlated with CNA, which suggest strong cis effects of CNA on mRNA abundance and underlines the important role of 20q involved in CRCs. For another thing, these up-regulated genes caused by CNA mainly resided on 20q11(20q11.23, 20q11.22 and 20q11.21) and 20q13(20q13.33, 20q13.32, 20q13.31, 20q13.13 and 20q13.12), which is in line with previous studies that show 20q11 and 20q13 are the most frequently amplified region.

Even though these genes on 20q demonstrated mRNA overexpression in great deal of CRCs, the proteins of these didn’t show the same result. Certain protein such as PIGU even demonstrated negative immunoreactivity in some CRC tissue, which corresponded to previous studies using integrated proteogenomic analyses that conclude that CNA–mRNA correlations were significantly higher than CNA–protein correlations for genes in all three groups (CNA, mRNA and protein)(P< 1.0×10-10, Kolmogorov–Smirnov test) [8]. All these studies, as well as ours, indicated many CNA-driven mRNA level increases do not translate into increased abundance of the corresponding proteins. In addition, we found that 3 genes(TM9SF4, PLAGL2 and POFUT1) with positive CNA–protein and mRNA correlations were not correlated with survival rate of CRC based on the data from the TCGA database. The paradox is that previous research has reported that TM9SF4, a key gene involved in cancer cell cannibalism, is a novel V-ATPase-interacting protein that regulates tumor pH alterations associated with drug resistance and invasiveness of colon cancer cells [16], and PALGL2, a prognostic factor, can serve as a tumor oncoprotein in the development and progression of colorectal cancer [17]. This highlights the fact that more work needs tobe done with the support of database. It is worth noting that not all genes with CNA can causemRNA over-expression; some even presented negative correlation, such as BCAS1and EDN3 [4, 15].Some researchers recommend that for the research on down-regulated genes in amplified chromosomal areas, which would be are obviously very interesting candidates to evaluate as putative tumor suppressors [18].

Interestingly, we found that these genes with mRNA overexpression showed a strong tendency to co-express, and what’s more, these genes with co-expression tend to lie adjacent to each other and formehysically adjacent gene cluster(2-7 genes) in the genome. Similar phenomena have been observed in a variety of species such as Drosophila,mice and humans [9]. Among the potential underlying mechanism, the sharing of regulatory elements such as transcription factors, promoters and enhancers is a key factor [9, 19, 20]. So in our study, an adjacent gene group including TM9SF4, POFUT1, PLAGL2, KIF3B and ASXL1 were subjected to Venn Diagrams analysis and found that they share 60 transcription factors,as supported by ChIP-Seq evidence. The enrichment analysis oftranscription that revealed that adjacent gene pairs and gene triplets are more likely to be modulated by same transcription factors. These results corresponded to previous studies that found that physical distance may play an important role in the co-expression of neighboring genes [19, 20].

As discussed above, these genes with short distance are prime candidates for sharing regulatory elements and presenting co-expression. When they separated of less than 1000bp, one classical example,a bidirectional promoter, can occur. Bidirectional promoters gene may be generated from two genes arranged in a head-to-head(adjacent 5’ ends) configuration with less than 1000 base pairs of intervening sequence, They are generally evolutionarily conserved, share transcription factors, presente higher GC content and often lack TATA boxes [21]. Expression of gene pairs driven by bidirectional promoters appeares to be correlated, as shown by PSENEN and U2AF1L4 [22], and PRR11 and SKA2 [23]. In our research, PLAGL2 and POFUT1 exhibited significant co-expression levels (r=0.84) and perfectly met the criteria described above, so we postulated that the co-expression of two genes is regulated by a bidirectional promoter.

It is noteworthy that many adjacent genes are coexpressed, but some including bidirectional pairs are antiregulated [24]. So in this article, we also explored another mechanism, the competitive endogenous RNA (ceRNA) hypothesis, which was recently aroused great interest in studies of human cancer. RNA transcripts that contain miRNA-binding sites can communicate with and indirectly regulate each other by competing specifically for shared miRNAs, in particular, resulting co-expression of RNA transcripts [25]. In our study, we found adjacent gene group, TM9SF4, POFUT1, PLAGL2, KIF3B and ASXL1 can share 9 common predicted miRNAs, especially the significantly correlated gene pair POFUT1 and PLAGL2 share common 52 microRNAs with experimental evidence, and these microRNAs can directly target PLAGL2 and POFUT1 and repress the expression of both genes in colon and rectal cancer, which indicate that ceRNA may be another important mechanism causing co-expression of these adjacent genes.

But the need to pay attention to is that although the co-expression of neighboring genes is a remarkable phenomenon, the mechanism behind that is as yet unclear [9, 19]. Many mechanisms can explain the co-expression of neighboring genes such as alteration of the chromatin structure, sharing of regulatory elements, chromatin domains, spatial organization and functional relationship of gene clusters [9, 20, 26]. Taking into account the content of the article, we only focused on three main mechanisms in this article. We hold that these mechanisms only can account for the part of the coexpression pattern and may be be able to provide some brief references for further research in this field.

Previous studies have also suggested that genes with co-expression characterization always participate in the same biological function. In our study, 6 adjacent genes with co-expression characterization in Figure 3(TM9SF4, PLAGL2, POFUT1, KIF3B, ASXL1, NOL4L) were chosen for gene-set pathway enrichment analysis. We found that these overexpressed adjacent genes caused by CNA in colorectal cancer can cooperatively participate in the same biological pathways associated with human cancers, including colorectal cancer.Additionally, we inhibited the expression of PLAGL2 and POFUT1 in SW620 cells by shRNA, the result found that PLAGL2 and POFUT1 both can be involved in *SRC* biological pathways. Recent studies have confirmed that several of the 6 genes discussed above are involved in human cancer, especially colorectal cancer., TM9SF4 is a novel V-ATPase-interacting protein that modulates tumor pH alterations associated with drug resistance and invasiveness of colon cancer cells [16], PLAGL2 induces epithelial-mesenchymal transition via Wnt/β-catenin signaling pathway in human colorectal adenocarcinoma [27] and overexpression of protein POUT1 accelerates hepatocellular carcinoma progression via the Notch signaling pathway [28]. It's remarkable that the function of V-ATPases in cancer was closely related to the wnt and notch signaling pathway [29], which indicateds to us that PLAGL2, POFUT1and TM9SF4 may cooperatively participated in the development human cancer. In addtition, KIF3B and ASXL1 alsohave the potential to participatein human cancer [30, 31].

In general, in this study we firstly identified that most frequently unregulated genes caused by CNA in colorectal cancer are all located on chromosome 20q.Upon investigation, we found that these genes demonstrated co-expression features and tended to be clustered within the same genomic neighborhoods, and then tried to explore the underlying mechanism by looking into the sharing of transcription factors, conserved bidirectional promoter and ceRNA hypotheses. But whether these mechanisms are synergistic or independent, more work needs to be done to better understand how these adjacent genes of 20q maintain co-expression in colorectal cancer. In addition, we found that co-expression of adjacent genes which cooperatively participatein the same biological pathways can be associated with the pathogenesis and development of human cancer, especially colorectal cancer; This means that there are now novel genomic targets for therapeutic intervention for colorectal cancer.

## MATERIALS AND METHODS

### The information and chromosomal distribution of genes located on 20q

The official symbol, official full name, location, cytoband and gene type of all genes on chromosome 20 conformed to human species assembly version GRCh38(NCBI and Ensemble). The gene organization, CpG islands, chromatin state annotation, transcriptional regulation and evolutionary conservation were visualized by tracks through UCSC Genome Browser(http://genome.ucsc.edu/). The data for adjacent gene analysis was downloaded from NCBI Map Viewer (http://www.ncbi.nlm.nih.gov/projects/mapview).(more details can be found in Supplementary Table 2).

### Somatic mutations and expression of genes

COSMIC (the Catalogue Of Somatic Mutations In Cancer) study on colon adenocarcinoma (Study Id COSU376)[32], cBioPortal [33] for Cancer Genomics (Colorectal Adenocarcinoma (TCGA, Provisional) 633 samples and Colorectal Adenocarcinoma (TCGA, Nature 2012) 276 samples) and Oncomine(GSE9348 and TCGA Colorectal) were used for somatic mutations and expression analysis of all genes [34].

### Protein expression and survival analysis

The protein expression of all genes were evaluated by The Human Protein Atlas project [35] and the expression data is derived from antibody-based protein profiling using immunohistochemistry. Survival analysis was performed by OncoLnc, a database that can link TCGA survival data to mRNA, miRNA, or lncRNA expression levels (http://www.oncolnc.org/).

### Evaluating CNA–mRNA, miRNA-mRNA, mRNA-mRNA and mRNA (transcription factor)-mRNA correlation

We downloaded mRNA and CNV data for colon and rectal cancer from the Cancer Genome Atlas(TCGA) portal [36],.Copy-number alterations and gene-expression data for all 276 samples were detected by Affymetrix SNP 6.0 microarrays, Illumina HiSeq, Agilent microarrays and RNA-Seq. 218 data setswith quantifiable CNA and mRNA measurements from TCGA(Supplementary Table 1) were used for CNA–mRNA evaluation and mRNA-mRNA evaluation of 3 genes (PLAGL2, POFUT1and STAU1). 244 data sets (Supplementary Table 4) were used for mRNA (transcription factor)-mRNA correlation evaluation. starBase v2.0 [37], which provide miRNA-target interactions processed from five miRNA prediction software programs and overlapped with CLIP-Seq data, was used to explore anti-correlation (pearson correlation: r<0, p-value<0.05) between miRNA and target genes across colon and rectal cancer. Additionally, relationships between genes were analyzed by GeneMANIA [38], a free public resource that offers a simple, intuitive web interface.

### Identification of regulatory transcription factors within promoters

Prediction of putative transcription factor binding sites was performed using software GeneCard [39] and the JASPAR database [40]. Gene Card provided a link to the Ensembl regulatory element, promoter length and a list of TFs (Transcription Factors) having TFBSs (Transcription Factor Binding Sites) within the promoter (based on ChIP-Seq evidence). Comprehensive gene set enrichment analysis for transcriptional machinery was carried out using the software Enrichr [41]. 2 libraries including position weight matrices (PWMs) from TRANSFAC and JASPAR, and transcription factor targets extracted from the Encyclopedia of DNA Elements(ENCODE) project were selected to identify transcription factors that are enriched for target genes within the input list.

### miRNA-mRNA intersection and ceRNA identification

TargetScan 7.1, which has been proven to have with higher sensitivity and precision than other target predicting programs, was used to predict biological miRNA-mRNA intersection [42]. The experimentally validated microRNAs were compiled from TarBase v7.0 [43] and miRTARBASE 6.0 [44], the predicted miRNA-target interactions were processed from five miRNA prediction software programs (TargetScan, PicTar, PITA, miRanda and RNA22) and overlapped with CLIP-Seq data compiled from Starbase v2.0; In addition, Starbase v2.0 was employed to predict competing endogenous RNA (ceRNA) pairs by integrating the interactions from potential microRNA targets (miRanda/mirSVR) which overlapped with CLIP-Seq data [37], and to select the genes which can maintain correlation with POFUT1and PLAGL2 in 14 human tumor tissues.

### Analysis of biological networks

All enrichment analyses of KEGG pathways were identified statistically with DAVID Bioinformatics Resources 6.8 [45],.STRING v10.5 [46] was used to provide a critical assessment and integration of protein-protein interactions.

### RT-qPCR

Total RNA was extracted by Trizol (Invitrogen) and reverse transcribed using ReverTra Ace qPCR RT Master Mix with gDNA Remover (TOYOBO).qPCR assays were performed by using KOD SYBR® qPCR Mix (TOYOBO) on LightCycler® 480II System (Roche) according to the manufacturer’s instructions. Treated samples were normalized to controls with the ^∆∆^Ct formula using GAPDH as an endogenous control. The primer used in this study can be found in Supplementary Table 6.

### Cell culture and transfection

The human colorectal cancer cell SW620 was maintained in L-15 medium supplemented with 10% fetal bovine serum, penicillin and streptomycin, and incubated at 37°C in 5% CO2. All Lentivirus shRNA were synthesized by Suzhou GenePharma(Suzhou, China). The shRNA of PLAGL2 and POFUT1 were transfected into the SW620 according to the manufacturer’s instructions. Cells were then collected and subjected to analysis after showing stability.

### Statistical analyses

Linear regression and correlation studies were performed using the software GraphPad Prism 7 (San Diego, California, USA). Linear regression finds the best line that predicts Y from X. Correlation computes the value of the Pearson correlation coefficient, r, and its value ranges from -1 to +1. All correlation analysis, including CNA–mRNA, mRNA-mRNA and mRNA (transcription factor)-mRNA, results (mean values±SD) were subjected to statistical analysis by *t*-test, in which p<0.05 was considered statistically significant. The software Venn Diagrams (http://bioinformatics.psb.ugent.be/) was used for calculating the intersection(s) of list of elements.

## SUPPLEMENTARY MATERIALS FIGURES AND TABLES


















