# Supplementary material for: Comprehensive bioinformatics analysis of the characterization and determination underlying mechanisms of over-expression and co-expression of genes residing on 20q in colorectal cancer
Source: Oncotarget. 2017 Aug 10;8(45):78642–59. doi: 10.18632/oncotarget.20204 (PMC5667988; doi:10.18632/oncotarget.20204)
Supplement: Supplementary file 1 [file oncotarget-08-78642-s001.pdf]

Comprehensive bioinformatics analysis of the characterization and determination underlying mechanisms of over-expression and co-expression of genes residing on 20q in colorectal cancer

SUPPLEMENTARY MATERIALS

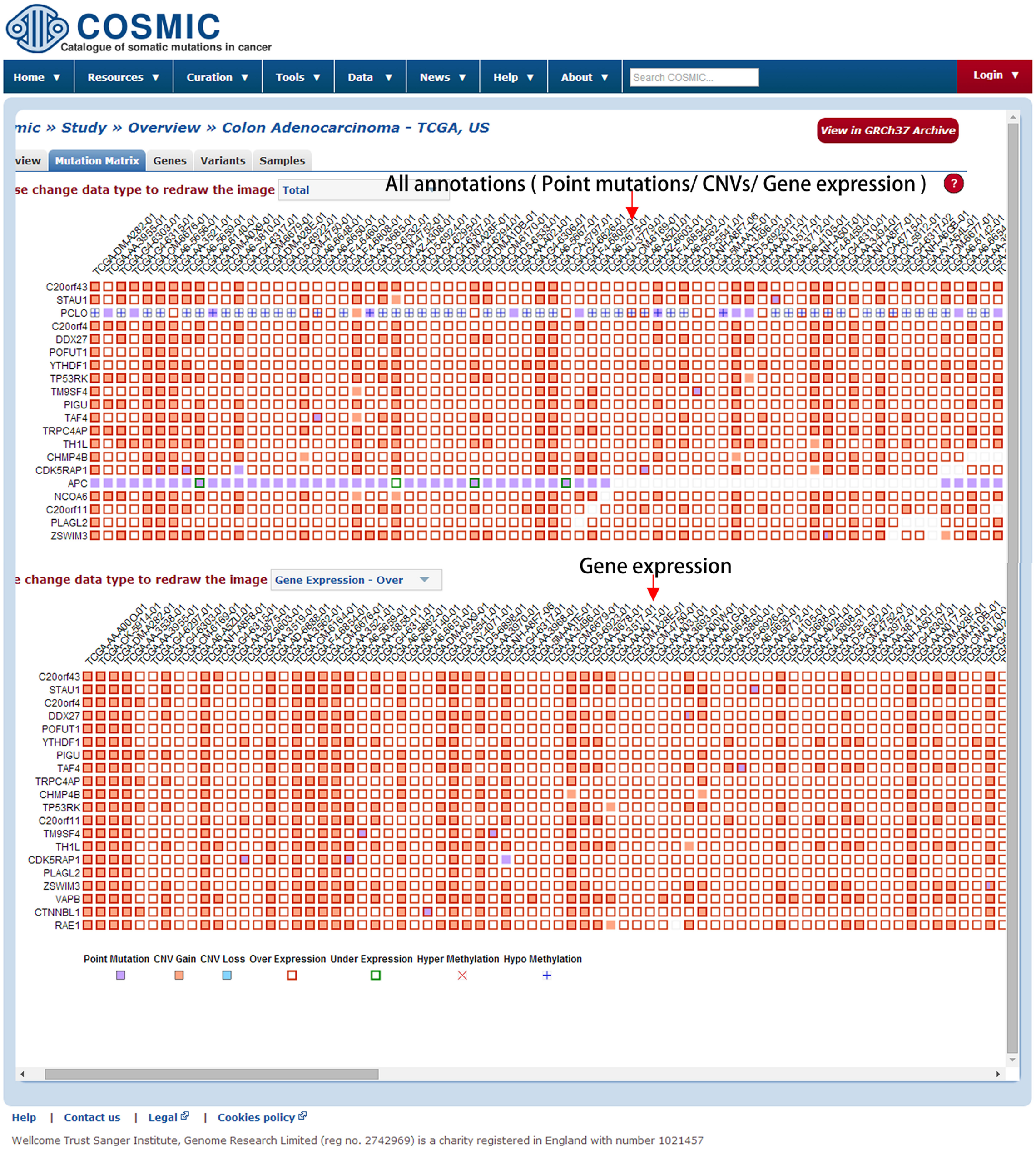

Supplementary Figure 1: The "Mutation Matrix" plot between genes and samples for study of colon adenocarcinoma - TCGA, US (Study Id: COSU376). (A/B) The chart contains a maximum of 20 genes (rows) and 175 samples (columns) with each box representing a Gene-Sample combination. A showed the result of all somatic mutations annotations including point mutations/ CNVs/ gene expression; B showed the result of over expression (more detail can be found in COSMIC).

Model ID	Model name	Score	Relative score	Start	End	Strand	predicted site sequence	
MA0470.1	E2F4	7.939	0.870532905441669	10	20	-1	gggcgggacca	
MA0470.1	E2F4	7.736	0.867318190252896	14	24	-1	gggcgggcggg	
MA0470.1	E2F4	5.330	0.829216689050002	18	28	-1	ccgcgggcggg	E2F4
MA0470.1	E2F4	5.869	0.837752312137433	49	59	1	gcgcgggcggc	
MA0470.1	E2F4	4.709	0.819382511058731	56	66	1	cggcgcgcagg	
MA0079.3	SP1	7.588	0.876606408292571	12	22	1	gtcccgcccgc	
MA0079.3	SP1	4.827	0.841869867390723	16	26	1	cgcccgcccgc	
MA0079.3	SP1	8.895	0.893049964597793	40	50	-1	gcaccgccccg	SP1
MA0079.3	SP1	1.804	0.803837066540926	47	57	-1	cgcccgcgcac	
MA0079.3	SP1	1.568	0.800867916358805	72	82	-1	cccgctcccag	
MA0079.3	SP1	8.846	0.892433488500657	74	84	-1	gccccgctccc	
MA0524.2	TFAP2C	11.981	0.931142159153826	20	31	-1	agcccgcgggcg	
MA0524.2	TFAP2C	11.967	0.930914652751575	20	31	1	cgcccgcgggct	
MA0524.2	TFAP2C	4.891	0.815926416870978	36	47	-1	ccgccccggccg	TFAP2C
MA0524.2	TFAP2C	3.925	0.800228475115656	36	47	1	cggccggggcgg	
MA0746.1	SP3	6.078	0.824044578045142	12	22	1	gtcccgcccgc	
MA0746.1	SP3	5.113	0.805040326911564	16	26	1	cgcccgcccgc	
MA0746.1	SP3	7.590	0.853821187075162	40	50	-1	gcaccgccccg	SP3
MA0746.1	SP3	5.604	0.814709847436524	72	82	-1	cccgctcccag	
MA0746.1	SP3	7.121	0.844584924088999	74	84	-1	gccccgctccc	
MA0516.1	SP2	11.877	0.885216720770935	12	26	1	gtcccgcccgcccgc	
MA0516.1	SP2	7.183	0.821515729193636	16	30	1	cgcccgcccgcgggc	
MA0516.1	SP2	10.966	0.872853788660386	36	50	-1	gcaccgccccggccg	
MA0516.1	SP2	5.932	0.804538749972672	43	57	-1	cgcccgcgcaccgcc	SP2
MA0516.1	SP2	11.661	0.882285443783143	70	84	-1	gccccgctcccagcg	
MA0162.2	EGR1	5.668	0.840879563047074	16	29	1	cgcccgcccgcggg	
MA0162.2	EGR1	8.526	0.871486349526517	37	50	-1	gcaccgccccggcc	EGR1
MA0162.2	EGR1	9.099	0.877622699649933	48	61	-1	gcgccgcccgcgca	
MA0162.2	EGR1	10.618	0.893889917516369	71	84	-1	gccccgctcccagc	
MA0471.1	E2F6	7.650	0.873888171746822	10	20	-1	gggcgggacca	
MA0471.1	E2F6	7.828	0.876514338522238	14	24	-1	gggcgggcggg	E2F6
MA0471.1	E2F6	5.272	0.838803763926937	49	59	1	gcgcgggcggc	

Comment: This type of analysis has a high sensitivity but abysmal selectivity. In other words: while true functional will be detected in most cases, most predictions will correspond to sites bound in vitro but with no function in vivo. A number of additional contraints of the analysis can improve the prediction; phylogenetic footprinting is the most common. We recommend using the ConSite service, which uses the JASPAR datasets.

The review Nat Rev Genet. 2004 Apr;5(4):276-87 gives a comprehensive overview of transcription binding site prediction

Supplementary Figure 2: The details about transcription factor binding sites including SP1, SP3, SP2, E2F4, E2F6, EGR1, TFAP2C predicted by the JASPAR database.

Supplementary Table 1: 218 TCGA data on CNA and mRNA of 8 genes used in article (All data coming from Comprehensive molecular characterization of human colon and rectal cancer [36].).

See Supplementary File 1

Supplementary Table 2: Sheet1: Coexpression between POFUT1, or STAU1 and all human genes mRNA expression (RNA_Seq_V2_RSEM) in colorectal cancer. Sheet 2: The chromosome distribution of all genes listed in sheet 1 (coexpression of POFUT1 and all human genes mRNA expression (RNA_Seq_V2_RSEM) in colorectal cancer, Pearson score ≥ 0.6). Sheet 3: All genes in Chromosome 20, the data download from NCBI Map Viewer. Sheet 4: 50 genes located within same CNV in 20q11.21 and 20q13.13 were selected for further co-expression analysis.

See Supplementary File 2

Supplementary Table 3: Sheet 1: The transcription factor binding sites within the promoter (based on ChIP-Seq evidence) the data compiled from GENECARDs, which provide a link to the Ensembl regulatory element, promoter length and a list of TFs (Transcription Factors). Sheet 2: The enrichment analyses of transcription category of 5 genes (ENCODE_TF_ChIP-seq_2015_ table). Sheet 3: The enrichment analyses of transcription category of 5 genes (TRANSFAC_and_JASPAR_PWMs_table). Sheet 4: The intersection transcription factors within promoters of 5 genes and their co-expression with POFUT1 (Pearson score). Sheet 5: The venn_result of Transcription Factors.

See Supplementary File 3

Supplementary Table 4: Sheet 1: The expression of PLAGL2, POFUT1, E2F4 and SP3 (All data coming from TCGA: Comprehensive molecular characterization of human colon and rectal cancer [36].). Sheet 2: The DNA sequence from 32207716 to 32207908 is highly conserved. Sheet 3: The location of PLAGL2 and POFUT1 on 20q in different gene database.

See Supplementary File 4

Supplementary Table 5: Sheet1: The predicted microRNAs by TARGETSCAN. Sheet 2: Experimentally validated microRNAs by TarBase v7.0 and miRTARBASE 6.0, and predicted miRNA-target interactions through Starbase which processed from five miRNA prediction software programs (TargetScan, PicTar, PITA, miRanda and RNA22) overlapped with CLIP-Seq data. Sheet 3: The intersection of miRNAs of PLAGL2 and POFUT1 gene. Sheet4: The venn_result of target genes predicted by TARGETSC.

See Supplementary File 5

Supplementary Table 6: The primers used in this article.

See Supplementary File 6

Supplementary Table 7: The database Starbase v2.0 predicted that the mRNA of POFTU1 and PLAGL2 show co-expression characteristics in 14 cancer types.

See Supplementary File 7

Supplementary Table 8: The gene-set of each gene (6 adjacent gene- TM9SF4, PLAGL2, POFUT1, KIF3B, ASXL1, NOL4 L-were selected for this study) (Pearson correlation coefficient≥0.2) was compiled from cBioPortal for Cancer Genomics (Colorectal Adenocarcinoma (TCGA [36]) 276 samples).

See Supplementary File 8
