# Supplementary material for: Comprehensive bioinformatics analysis of the characterization and determination underlying mechanisms of over-expression and co-expression of genes residing on 20q in colorectal cancer
Source: Oncotarget. 2017 Aug 10;8(45):78642–59. doi: 10.18632/oncotarget.20204 (PMC5667988; doi:10.18632/oncotarget.20204)
Supplement: Supplementary file 7 [file oncotarget-08-78642-s007.docx]

Supplementary Table 6: The primers used in this article

The primers were as follows:

1. PLGAL2 (forward primer GAGTCAAGTGAAGTGCCAATGT, reverse primer TGAGGGCAGCTATATGGTCTC)
2. POFTU1 (forward Primer AACCAGGCCGATCACTTCTTG, reverse primer GTTGGTGAAAGGAGGCTTGTG)
3. GAPDH (forward primer ACAACTTTGGTATCGTGGAAGG, reverse primer GCCATCACGCCACAGTTTC)
4. SRC (forward primer GAGCGGCTCCAGATTGTCAA, reverse primer CTGGGGATGTAGCCTGTCTGT).
