# Supplementary material for: Comprehensive bioinformatics analysis of the characterization and determination underlying mechanisms of over-expression and co-expression of genes residing on 20q in colorectal cancer
Source: Oncotarget. 2017 Aug 10;8(45):78642–59. doi: 10.18632/oncotarget.20204 (PMC5667988; doi:10.18632/oncotarget.20204)
Supplement: Supplementary file 8 [file oncotarget-08-78642-s008.docx]

| **cancerType** | **sampleNum** | **r** | **rank** | **p-value** | **FDR** |
| --- | --- | --- | --- | --- | --- |
| Urothelial bladder cancer (BLCA) | 230 | 0.60747 | 5245 | 1.37E-24 | 3.54E-22 |
| Breast cancer (BRCA) | 1106 | 0.43935 | 63380 | 0 | 0 |
| *Colon and Rectal adenocarcinoma (CRC)* | *362* | *0.91138* | *22* | *0* | *0* |
| Glioblastoma multiforme (GBM) | 167 | 0.5293 | 31405 | 1.93E-13 | 8.29E-12 |
| Head and neck squamous cell carcinoma (HNSC) | 467 | 0.40547 | 90375 | 6.60E-20 | 9.86E-19 |
| Chromophobe renal cell carcinoma (KICH) | 91 | 0.88272 | 52 | 6.13E-31 | 1.59E-26 |
| Clear cell kidney carcinoma (KIRC) | 579 | 0.45145 | 107080 | 2.03E-30 | 2.56E-29 |
| Acute Myeloid Leukemia (LAML) | 173 | 0.41574 | 118135 | 1.28E-08 | 1.47E-07 |
| Lung adenocarcinoma (LUAD) | 548 | 0.50025 | 14378 | 4.83E-36 | 4.54E-34 |
| Lung squamous cell carcinoma (LUSC) | 533 | 0.5784 | 5851 | 0 | 0 |
| Ovarian serous cystadenocarcinoma (OV) | 266 | 0.67191 | 371 | 2.74E-36 | 9.96E-33 |
| Cutaneous melanoma (SKCM) | 356 | 0.29756 | 351664 | 1.04E-08 | 3.98E-08 |
| Papillary thyroid carcinoma (THCA) | 558 | 0.34511 | 371750 | 4.74E-17 | 1.72E-16 |
| Uterine corpus endometrial carcinoma (UCEC) | 163 | 0.63755 | 19249 | 5.65E-20 | 3.96E-18 |

Supplementary Table 7: The database Starbase v2.0 predicted that the mRNA of POFTU1 and PLAGL2 show co-expression characteristics in 14 cancer types

***63 genes which can maintain correlation with POFUT1and PLAGL2 in a variety of human tumor tissues were selected for protein-protein interactions by STRING v10.5**

ABR

ANKFY1

APOLD1

AREL1

BAX

BCL2L11

BLOC1S3

C22orf29

CAPN7

CBX5

CEP170B

CNNM3

DNAJC27

EEF1GP5

GPAM

GPCPD1

GUCD1

H2AFX

ITGA2

KDELC2

KIAA0247

KIF1B

KLHL21

LARP1

LASP1

LBH

LRRC20

MAP3K9

MAVS

MKNK2

MPI

MRC2

MXI1

MYO1D

N4BP1

PHC2

PKD1

PLXNA1

PLXNA3

POLR1A

RAB35

RELA

RHOC

RNF24

RPS6KA3

SEMA4B

SHROOM3

SNHG16

SNX12

SOGA1

SRC

STRN4

TET1

TMEM25

TOLLIP

UNKL

WDR35

WDTC1

WHSC1

ZNF500

ZNF609

ZNF827

ZNFX1
